# Peritoneal metastasis histological grade independently predicts outcome in pseudomyxoma peritonei treated with curative surgery

**DOI:** 10.1007/s12094-025-04112-8

**Published:** 2026-01-20

**Authors:** Awen Hasan, Bipasha Chakrabarty, Christopher Kearsey, Raghavendar Nagaraju, Malcolm Wilson, Andrew Renehan, Rebecca Fish, Paul Sutton, Jonathan Wild, Chelliah Selvasekar, Hamish Clouston, Jorge Barriuso, Sarah Theresa O’Dwyer, Omer Aziz

**Affiliations:** 1 Division of Cancer Sciences, Faculty of Biology, Medicine and Health, University of Manchester, Manchester, UK; 2 Colorectal and Peritoneal Oncology Centre (CPOC) at The Christie NHS Foundation Trust, Wilmslow Road, Manchester, M20 4BX UK

**Keywords:** Pseudomyxoma peritonei, Appendiceal mucinous neoplasm, Peritoneal metastasis, Histological grade, Classification, Prognosis

## Abstract

**Background:**

Pseudomyxoma Peritonei is characterised by peritoneal metastasis from appendiceal mucinous neoplasms (AMN). The PSOGI classification (2016) categorises PMP into acellular mucin (AM), low-grade mucinous carcinoma peritonei (LGMCP), and high-grade mucinous carcinoma peritonei (HGMCP). This study aimed to determine long-term prognosis based on this classification.

**Materials and methods:**

Pathology review from PMP patients with AMNs undergoing cytoreductive surgery and heated intraperitoneal chemotherapy (CRS + HIPEC) with curative intent over a 15-year period (2006–2021) was undertaken. Patients underwent standardised surveillance. Cox proportional hazards regression models, log-rank test, and Kaplan–Meier method were used to assess overall (OS) and disease-free survival (DFS) based on histopathological peritoneal metastasis grade. DFS was only calculated for patients who had a complete cytoreduction.

**Results:**

290 PMP patients were identified (AM = 34%, LGMCP = 59%, HGMCP = 7%) with median follow-up of 49 months. Median age was 59 years (range: 22–79), M: F of 1:2.5, peritoneal cancer index median of 18 (range: 0–39). Univariate OS hazard ratio (HR) is 2.75 for LGMCP vs AM (95% CI: 1.05 –7.21, *p* < 0.040) and 14.29 for HGMCP vs AM (95% CI: 3.92– 52.11, *p* < 0.001). DFS HR = 5.15 for LGMCP (95% CI: 2.19–12.10, *p* < 0.001) and 4.16 for HGMCP (95% CI: 1.03–16.80, *p* < 0.045) with an overall peritoneal metastasis *p* value < 0.001. Multivariate OS analysis showed that peritoneal histology for HGMCP remained a significant predictor of poor prognosis for OS (HR: 5.54, 95% CI: 1.32–23.25, *p* = 0.019), whilst LGMCP did not demonstrate a significant association (HR: 1.59, 95% CI: 0.59–4.26, *p* = 0.359).

**Discussion:**

Peritoneal metastasis histopathological grade predicts outcome for patients with PMP from AMN following CRS + HIPEC independent of primary histology.

## Introduction

Pseudomyxoma Peritonei (PMP) is a rare clinicopathological condition, characterised by peritoneal dissemination and progressive accumulation of mucinous tumour. PMP is associated with solid peritoneal tumour implants, mucinous fluid accumulation, abdominal distension, and small bowel compression leading to intestinal obstruction, malnutrition, cachexia, and mortality if left untreated [1]. Appendix tumours can perforate or grow through its wall resulting in dissemination of neoplastic cells and mucin into the peritoneal cavity, presenting the characteristic mucinous ascites seen in PMP. The WHO classifies these primary tumours of the appendix into low- and high-grade AMNs (LAMN and HAMN) [2]. PMP has an estimated annual incidence of 1–2 per million [3].

Aggressive locoregional treatment involves cytoreductive surgery (CRS) to remove all visible tumour followed by hyperthermic intraperitoneal chemotherapy (HIPEC) to treat any microscopic residual neoplastic cells. This procedure is accepted widely as the standard care for PMP. The overall survival in patients treated with CRS + HIPEC is 63% at 10 years and 59% at 15 years, with the higher pathological grade category of the peritoneal disease thought to result in poorer outcomes [3–6].

**Fig. 1:**
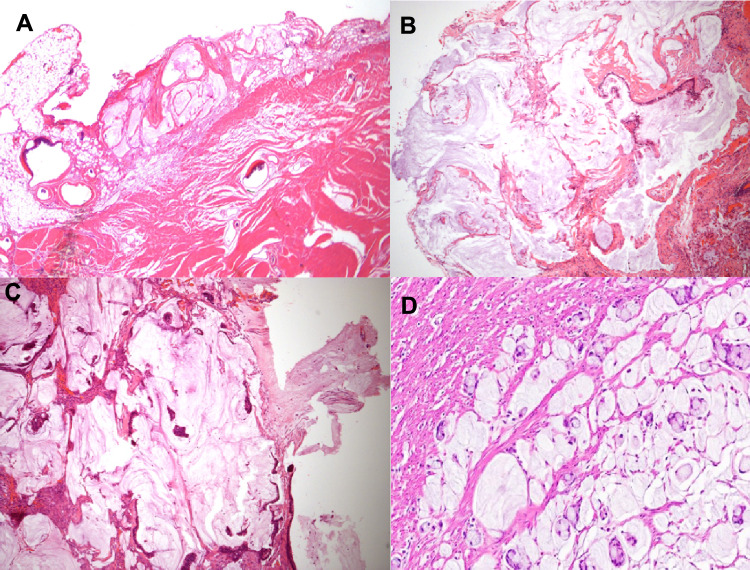
**A** PMP derived from acellular mucin (AM) secondary to AMN. **B** Low-grade mucinous carcinoma peritonei (LGMCP), peritoneal metastasis with low-grade histological features. **C** High-grade mucinous carcinoma peritonei (HGMCP). **D** High-grade mucinous carcinoma peritonei with signet ring cells (HGMCP-S)

There have been several histological classification systems used to grade peritoneal pathology in PMP, including Ronnet et al*.* [7], Bradley et al*.* [8], and, most recently, the Peritoneal Surface Oncology Group International-PSOGI [9]. The PSOGI classification system categorises the peritoneal pathology of PMP into Acellular mucin (AM), low-grade mucinous carcinoma peritonei (LGMCP), and high-grade mucinous carcinoma peritonei, with or without signet ring cells (HGMCP/HGMCP-S) shown in Fig. 1.

Whilst there have been studies that have reported on the impact of peritoneal pathology category on outcomes from CRS + HIPEC for PMP, few have had classified the peritoneal pathology using the PSOGI (2016) system and had access to the primary AMN in all cases [10, 11]. Being certain the primary appendix tumour was an AMN is important, because other more aggressive tumours of the appendix (such as adenocarcinomas) can mimic their peritoneal pathology, confounding results. This study aims to report long-term outcomes for a cohort of patients with PMP from confirmed AMNs classified by a specialist pathologist using the PSOGI (2016) system at a high-volume peritoneal tumour service.

## Materials and methods

### Patients

A review of a prospectively maintained database was undertaken to identify 2079 patients with appendix tumours referred to a UK peritoneal tumour centre between 2006 and 2021, of which 1259 underwent surgery. 692 of these patients had AMNs and had their cases reviewed. This study was approved by the local regional ethics committee.

### Selection

Six hundred and ninety-two patients had their external specimens reviewed by a pathologist specialising in appendix tumours, and each case discussed at a peritoneal tumour multi-disciplinary team (MDT) comprising peritoneal tumour surgeons, radiologist, pathologist, medical oncologist, and clinical nurse specialists. 290 patients who had AMNs and established peritoneal disease (PMP) underwent CRS and HIPEC with curative intent. Patients were excluded if they had AMNs without established peritoneal disease.

### Operative technique

The operative technique used for CRS + HIPEC with Mitomycin C (35 mg/m^2^ at 42 °C over 90 min infusion) at our institution has previously been described [10].

### Pathological classification

The WHO (2019) classification system [11] was used to re-categorise the primary AMNs into low-grade (LAMN) and high-grade (HAMN). The PSOGI [12] classification system was used to re-categorise peritoneal metastases into Acellular mucin (AM), Low-grade mucinous carcinoma peritonei (LGMCP), and High-grade mucinous carcinoma peritonei without or with signet ring cells (HGMCP/HGMCP-S).

### Surveillance

Following CRS + HIPEC patients had CT abdomen and pelvis with oral and intravenous contrast accompanied by tumour markers (CEA, Ca19.9 and Ca125) 6 monthly for 2 years and at years 3, 4, 5, 8, and 10 [10].

### Study parameters

Patient age, sex, peritoneal cancer index (PCI), completeness of cytoreduction (CC) score, and the histological grade of the peritoneal disease were accounted for. The PCI quantifies peritoneal disease volume and distribution ranging from 0 to 39 [12]; our series contained seven cases classified as PCI-0 at time of surgery. During pathological evaluation, these specimens were found to have a degree of disease, with two cases of LMCP and five cases of AM; these cases were retained in the series to ensure transparency. The CC score records any residual or serosal mucinous material at end of procedure: CC0 = no disease, CC1 =  < 2.5 mm or a film of mucinous material diffuses on the small bowel serosa (no solid implants), CC2 = 2.5–25 mm, and CC3 =  > 25 mm [10]. Overall survival (OS) was calculated based the date of CRS + HIPEC to the date of last follow-up or date of death. Disease recurrence was defined as peritoneal abnormality seen on a surveillance CT scan which was reported as recurrent disease and confirmed after discussion at a specialist peritoneal MDT meeting. Timepoint for recurrence was marked as the date of the indicative CT scan.

### Statistical analysis

Overall survival (OS) and disease-free (DFS) analysis was carried out using the Kaplan–Meier method [13]. Log-rank test was used to evaluate statistical significance between peritoneal metastasis histopathological grades.

## Results

We identified 290 patients with AMN, of which 270 patients were diagnosed with LAMN, whilst 20 had HAMN. The PSOGI classification of peritoneal metastases in the cohort included: AM in 97 patients and LGMCP in 171, our series only contained one case of HGMCP-S, and thus, this was grouped with the HGMCP totalling 22 cases. The median follow-up duration was 49 months. The Cohort summary is outlined in (Table. 1).

## Patient survival outcomes

### Overall survival at 5- and 10-year periods

The patient overall survival probability at 5 years was 87%, declining to 75% at 10 years in our cohort.

### Overall survival (OS) based on PCI score

**Fig. 2:**
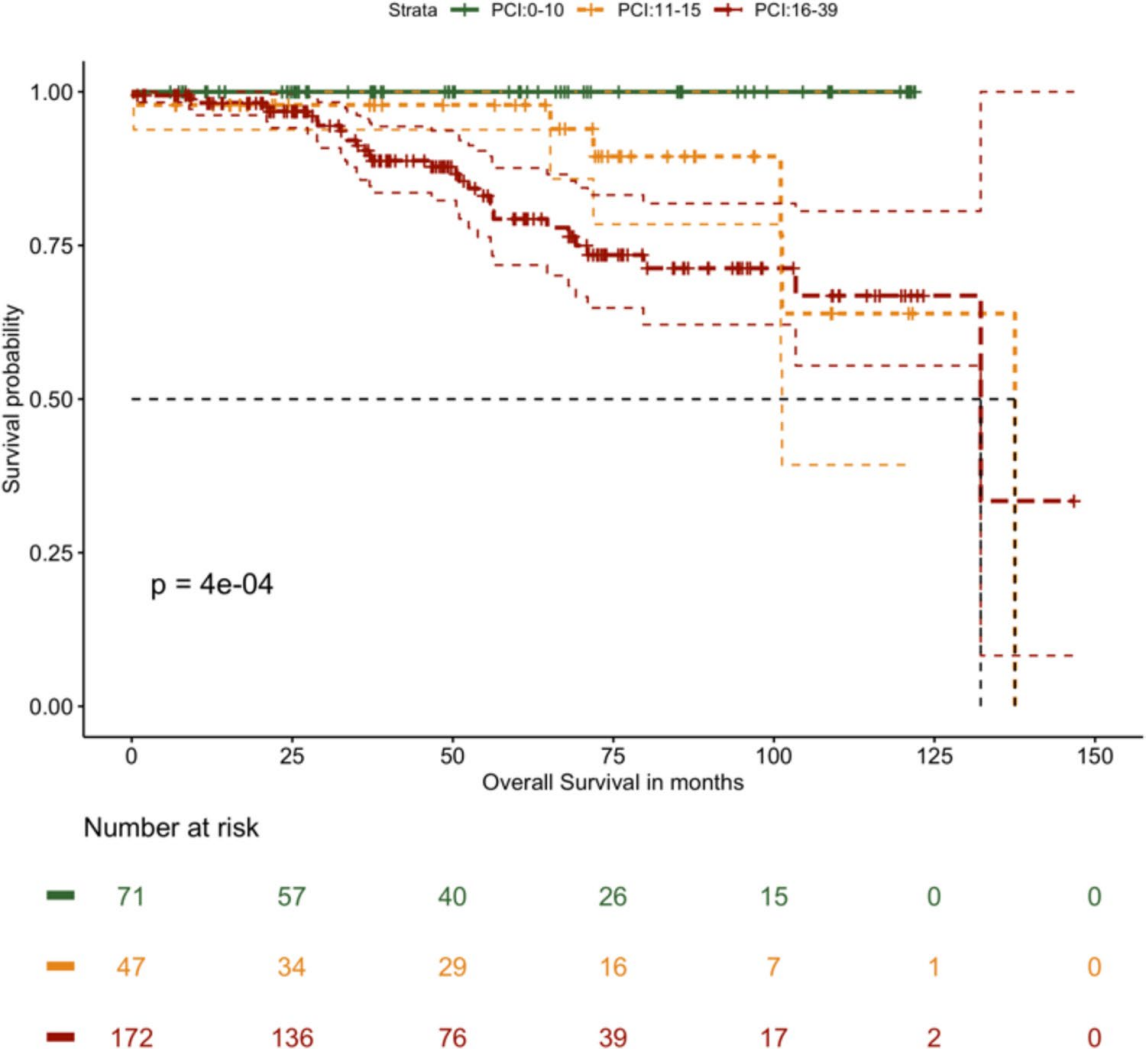
KM curves for: overall survival outcome based on peritoneal cancer index (PCI), categorised into three subgroups: PCI: 0–10, PCI: 11–15, and PCI: 16–39

Kaplan–Meier analysis (Fig. 2) demonstrated a statistically significant difference in OS between the PCI groups (*p* < 0.0004). Patients with PCI score 0–10 had the most favourable survival outcomes, with a probability close to 1 over the observed time. In contrast, patients with higher PCI scores 11–15 and 16–39 showed progressively lower survival probabilities, with the highest PCI groups having the worst outcomes. The medians of OS were not reached. At 5 years, survival rates were notably lower for patients in the PCI 16–39 group compared to those with PCI 0–10, emphasising the strong association between higher PCI scores and decreased overall survival. The number at risk for each group is displayed at various time points, highlighting the consistent decline in survival with increasing PCI score.

Cox proportional hazards regression analysis was conducted to assess the impact of PCI on disease-free survival (DFS) in patients with CC0-CC1. The model approached statistical significance with a Chi-square statistic of 3.678 and a *p* value of 0.055. The hazard ratio was 1.893 (95% CI: 0.969–3.700, *p* = 0.062), suggesting a trend towards increased risk of recurrence with higher PCI scores. However, the confidence interval crossed 1, indicating that the result was not statistically significant (*p* = 0.062).

### OS based on completeness of cytoreduction (CC) score

The long-rank test revealed a statistically significant difference in OS across CC scores, with a Chi-square statistic of 55.84 and a corresponding *p* < 0.001. The HR for mortality were 11.40 (1.52–85.24) *p* < 0.018 for CC1, 51.66 (6.58–405.81) *p* < 0.001 for CC2, and 97.68 (10.85–879.58) *p* < 0.001 for CC3, indicating progressively higher risks of mortality with increasing CC scores.

### Overall survival based on peritoneal histological category

**Fig. 3:**
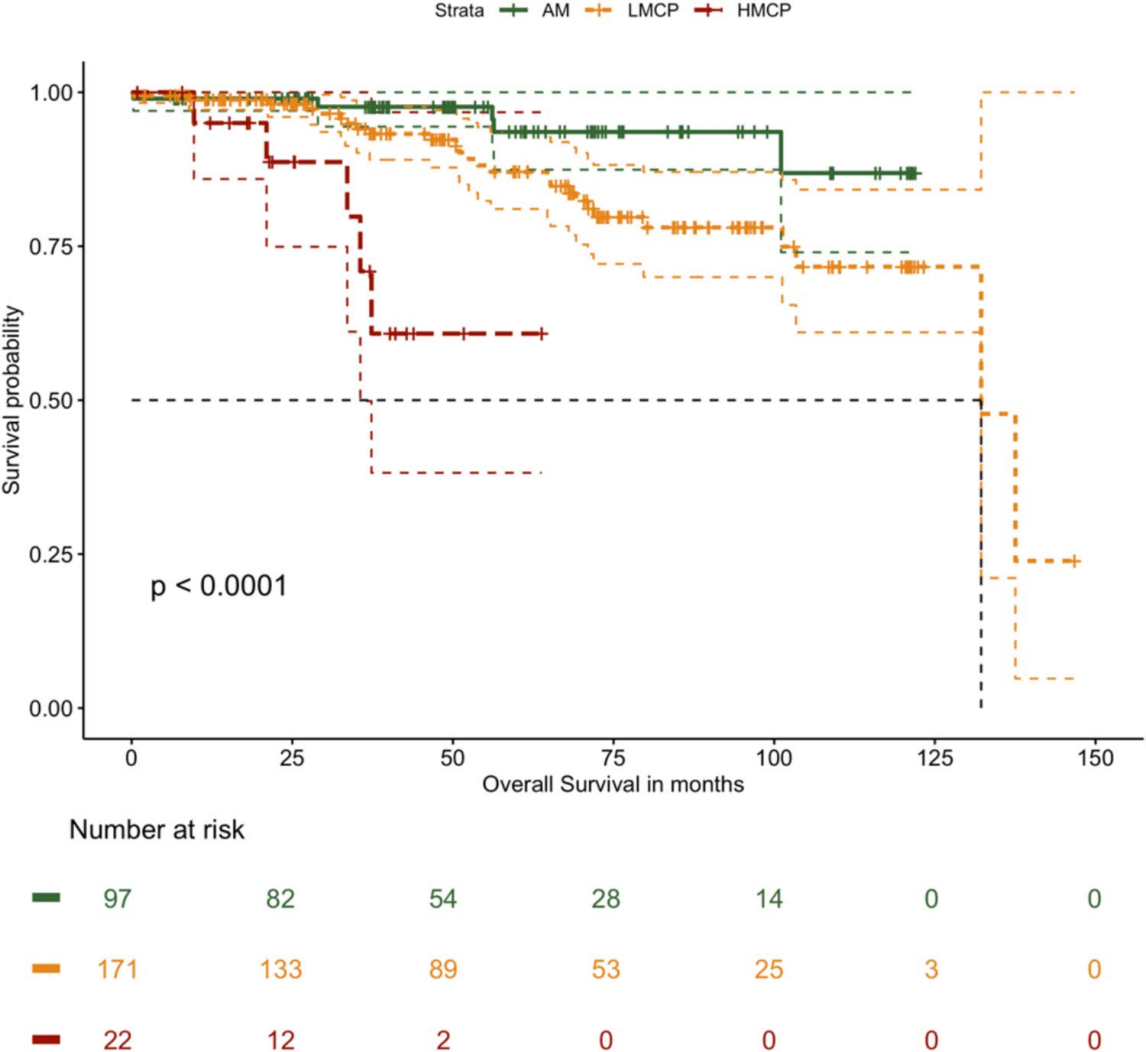
Patient overall survival outcome based on peritoneal metastasis histology. Acellular mucin (AM), low-grade mucinous carcinoma peritonei (LGMCP), high-grade mucinous carcinoma peritonei (HGMCP)

KM curves based on peritoneal pathology grade are shown in Fig. 3. AM patients had the most favourable outcome followed by LGMCP and HGMCP, who had the worst survival outcome. The Log-rank statistic was 22.2 with associated *p* value of < 0.001. The cox proportional hazard (HR) (95% CI) analysis showed that LGMCP had an HR of 2.75(1.049–7.211), with a statistically significant *p* < 0.040. HR for HGMCP was 14.29 (3.92–52.07), *p* < 0.001. OS medians not reached .

**Table 1:** Cohort summary

	AMN undergoing CRS + HIPEC (*n* = 290) n (%)
Sex	
Female	208 (72)
Male	82 (28)
Median age time of diagnosis (range)	59 (22–79)
Completeness of cytoreduction	
CC-0	85 (29)
CC-1	161 (55)
CC-2	28 (10)
CC-3	16 (6)
PCI median	18 (0–39)
Primary appendix tumour	
LAMN	270 (93)
HAMN	20 (7)
Peritoneal pathology	
AM	97 (34)
LGMCP	171 (59)
HGMCP	22 (7)
Peritoneal pathology originating from LAMN	
AM	93 (34)
LGMCP	161 (60)
HGMCP	16 (6)
Peritoneal pathology originating from HAMN	
AM	4 (20)
LGMCP	10 (50)
HGMCP	6 (30)
Median follow-up in months	49 (0.26–146)
Median cohort follow-up in months	52

### Disease-free survival based on peritoneal histological category

**Fig. 4:**
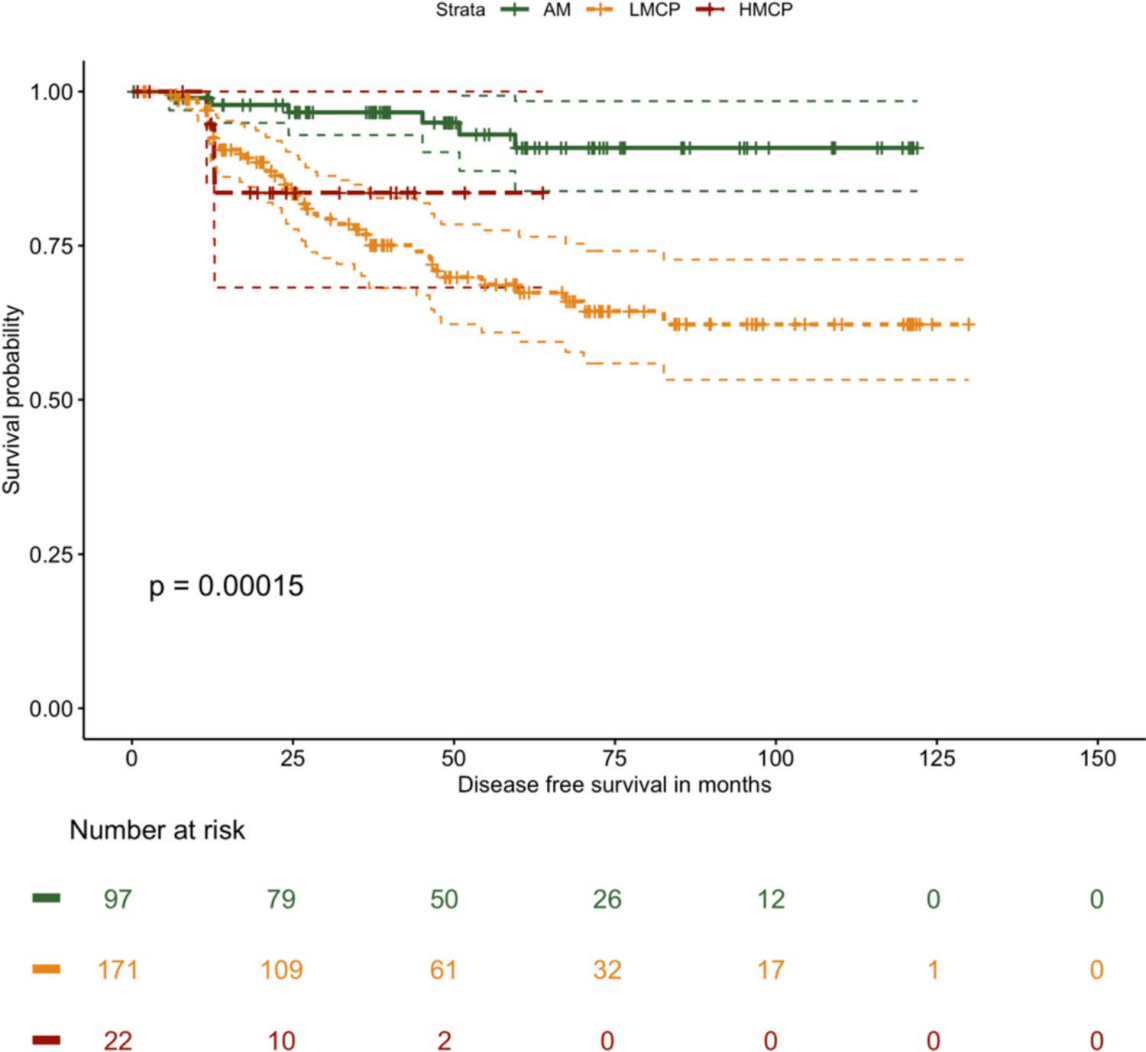
Patient disease-free survival based on peritoneal pathology: acellular mucin (AM), low-grade mucinous carcinoma peritonei (LGMCP), high-grade mucinous carcinoma peritonei (HGMCP)

The log-rank test revealed statistically significant difference, with a chi-statistic of 17.6 and a corresponding *p* value of < 0.001. The HR for recurrence was 5.15 (2.19–12.08) for LGMCP compared to AM and 4.16 (1.03–16.82) with a *p* < 0.045 for HGMCP compared to AM, demonstrating a statistically significant difference between peritoneal disease subgroups (Fig. 4).

### Overall survival univariate and multivariate analyses

The log-rank test (Mantel–Cox) was performed to identify statistically significant factors affecting overall survival, including complete cytoreduction (CC), peritoneal cancer index (PCI), and peritoneal metastasis histological subgroups. A multivariate Cox proportional hazards regression analysis was subsequently conducted (Table 2). Our analysis revealed the CC score was significantly associated with overall survival (*p* < 0.001).

**Table 2:** Overall survival analysis using univariate and multivariate (Cox model), featuring complete cytoreduction index at operation, peritoneal cancer index, histological category/grade of peritoneal metastasis, age, and gender

Univariate							Multivariate			
Variable	Category	*P* value	HR	L95% CI	U95% CI	Log-rank *P* value	*P* value	HR	L95% CI	U95% CI
Complete cytoreduction at operation (CC)		**< 0.001**	–	–	–	**< 0.001**	** < 0.001**	–	–	–
	CC1 vs CC0	**0.018**	11.396	1.524	85.236		0.228	3.490	0.458	26.598
	CC2 vs CC0	**< 0.001**	51.661	6.577	405.810	–	**0.012**	15.153	1.829	125.520
	CC3 vs CC0	**< 0.001**	97.683	10.848	879.579	–	**0.008**	21.181	2.212	202.806
Peritoneal cancer index (PCI)		**< 0.001**	–	–	–	**< 0.001**				
	0–10	0.404	–	–	–	–	0.990	–	–	–
	11–15	0.902	66,724.171	0.000	3.494	–	0.895	15,646.072	0.000	5.659
	16–39	0.897	121,938.482	0.000	6.376	–	0.895	16,070.453	0.000	5.803
Peritoneal metastasis histology		**< 0.001**	–	–	–	**< 0.001**	0.051			
	LGMCP vs AM	0.040	2.750	1.049	7.211	–	0.359	1.588	0.591	4.263
	HGMCP vs AM	**< 0.001**	14.298	3.923	52.111	–	0.019	5.536	1.318	23.250
Primary tumour of appendix histology	LAMN vs HAMN	0.556	0.047	0.000	352.925	0.309	0.953	0.000	0.000	2.306e + 163
Sex	Male vs Female	0.817	1.091	0.523	2.272	0.817	0.929	0.966	0.448	2.082
Age		1.000	–	–	–	**< 0.001**	0.069	1.031	0.998	1.066

*HR* Hazard ratio, *LCI* lower confidence interval, *UCI* upper confidence interval

In the univariate analysis, patients with CC1 showed a hazard ratio (HR) of 11.4 (95% CI: 1.5–85.2, *p* = 0.018). However, in the multivariate analysis, the association was not statistically significant (HR: 3.5, 95% CI: 0.5–26.6, *p* = 0.228). The risk increased significantly in the CC2 group (univariate HR: 51.7, 95% CI: 6.6–405.8, *p* < 0.001; multivariate HR: 15.2, 95% CI: 1.8–125.5, *p* = 0.012), indicating a worse prognosis. As anticipated, patients with CC3 had the least favourable outcomes, with a univariate HR of 97.7 (95% CI: 10.8- 879.6, *p* < 0.001) and a multivariate HR of 21.2 (95% CI:2.2–202.8, *p* = 0.008).

Age was borderline significant, with a multivariate HR of 1.031 (95% CI: 0.998–1.066, p = 0.069).

We found that histological grade of peritoneal metastasis plays a role in survival. In the univariate analysis, patients with HGMCP had a significantly worse prognosis compared to AM, with an HR:14.3 (95% CI:3.9–52.1, *p* < 0.001). The multivariate analysis also confirmed this significant association HR:5.5 (95% CI:1.3–23.3, *p *= 0.019). Conversely, LGMCP group did not show a significant association with survival compared to AM in the multivariate analysis HR:1.6 (95% CI: 0.6–4.3, *p* = 0.359).

Multivariate analysis confirmed the significant impact of CC score on survival, with CC2 HR:15.2 (95% CI:1.8–125.5, *p* < 0.012) and CC3 HR:21.2 (95% CI:2.2–202.8, *p* = 0.008) being a strong predictor of poor prognosis. The primary appendix tumour did not demonstrate a significant association with survival HR:0.00 (95% CI:0.00–2.306e + 163, p = 0.953); the wide confidence interval is likely due to the large dispersity in sample size between the LAMN (*n* = 270) vs HAMN (*n* = 20) groups, leading to the presented estimates. Additionally, sex was not significantly associated with increased hazard, yielding an HR: 1.9 (95% CI: 0.4–2.1, *p* = 0.008).

## Discussion

This study of 290 PMP patients with confirmed peritoneal metastases homogenously classified by specialist pathologists using the PSOGI 2016 system, all arising from their matched AMNs, represents one of the largest series of its kind in the reported literature. The most common peritoneal pathology grade was LGMCP (59%) followed by AM (34%) and HGMCP (7%). The impact of the volume of disease (PCI) and clearance at surgery (CC score) on long-term outcomes (OS and DFS) for this cohort were as expected and has been previously reported [14]. Lower PCI and lower CC scores were associated with better outcomes, validating the dataset and demonstrating that surgical clearance of peritoneal disease is one of the most important determinants of success. This study has found that peritoneal pathology (classified using the PSOGI 2016 system) independently predicted outcome in patients undergoing CRS + HIPEC with curative intent for PMP arising from AMNs. AM patients had the best outcomes followed by LGMCP and HGMCP.

Although peritoneal mucinous deposits originate from the primary tumour, the pathological grade of the appendix primary does not always match that of the peritoneal disease. In this cohort, peritoneal metastasis that originated from primary LAMNs consisted of AM (34%), LGMCP (60%), and HGMCP (6%), whilst the peritoneal metastasis that originated from primary HAMNs consisted of AM (20%), LGMCP (50%), and HGMCP (30%). It is recognised and previously reported that pathological discordance between the primary tumour of the appendix and peritoneal disease can occur, and that peritoneal metastasis pathological grade determines survival [15, 16].

This study has also identified PMP patient groups that should be the focus of further research. First, we found that the majority of the primary AMNs were LAMNs (93%) compared to HAMNs (7%), which are a rarer entity. Whether HAMNs have a poorer long-term outcome compared to LAMNs remains unknown. Interestingly, within our cohort, we found that HAMNs had a higher proportion of HGMCP (30%) compared to LAMNs (6%). Validating these findings will require access to more patients with PMP arising from HAMNs. Second, it may be that whilst we have classified peritoneal pathology into discrete groups (AM, LGMCP, HGMCP), there is in fact a spectrum of peritoneal disease requiring further sub-categorisation. For example, there are some LGMCP patients that behave more like HGMCP, suggesting that there may be an intermediate group. Addressing these questions will require increased sample size through multi-centre collaboration and homogenisation of pathology reporting. In parallel, comprehensive variant mutation mapping will be essential to identify potential driver mutations and molecular pathways underlying these transitions observed within this retrospective study, in addition to review of pathological characteristics in this intermediate spectrum group.

To date, whilst the impact of histopathological markers on PMP outcomes has been the focus of research, there remains an absence of comprehensive understanding of the specific genomic drivers in AMNs and their peritoneal metastases. Known mutations, such as KRAS and GNAS, provide valuable insight into tumour biology and commonly found in LGMCP [17] are considered as early events in tumorigenesis, whereas alterations in PIK3CA, SMAD4, and TP53 have been associated with high-grade transformation and worse prognosis [18]. However, further research is needed to identify additional potential molecular drivers that reflect the different characteristics of the disease across the categories. This will only be possible through DNA and RNA sequencing of matched tumours, as well as the analysis of expressed proteins. https://pmpnet.eu is an accelerator that aims to address this through prospective biobanking of samples in PMP patients in 3 countries using standardised operating procedure. In-vitro and in-vivo models also offer the opportunity to identify new treatment targets for these patients.

Recent studies have reported the limitations of circulating tumour DNA (ctDNA) in PMP. In a multi-centre study of 21 patients, ctDNA was found to be undetectable in peripheral blood, although KRAS and GNAS mutations were present in tumour tissue, suggestive of minimal systemic tumour DNA release [19]. Furthermore, a study of three PMP patients with acellular mucin, KRAS-mutated cell-free DNA was identified in mucin samples but not in pre- or post-surgery plasma, [20]. This suggests a lack of detectable ctDNA shed into the bloodstream in PMP and that mucin cfDNA could provide information to routine pathological assessment. Its potential as a relevant biomarker for disease monitoring and risk stratification requires further exploration across a larger cohort of varying pathological grades.

This study had several limitations. First, AMNs are rare tumours, resulting in a relatively small sample size used in this study, collected over a period of time. Second, this represented a highly selected group of patients who were able to undergo CRS/HIPEC in a standardised manner at our institution. There are patients who present with very advanced disease for whom surgery is not an option and little information is available on the impact of systemic anticancer treatment (SACT) on their OS and DFS. Traditionally SACT is not offered to those with LGMCP and reserved mainly for HGMCP patients within this group. Third, there was a large representation of primary low-grade appendiceal mucinous neoplasms within our cohort due to high-grade appendiceal mucinous neoplasms being less common. Fourth, we acknowledge the small sample size in the HGMCP group, however, feel that our findings provide an important insight into the outcomes for this poorly understood sub-group that should be the focus of future research. Rigorous data collection and log-rank test were used to maximise the validity of our findings. Finally, the pathological sampling of specimens for these patients can be challenging, with a large number of samples from which a representative group is obtained. Our specialist pathologists have developed over 2 decade experience in doing this.

## Conclusion

This study of 290 PMP patients is one of the largest cohorts of appendiceal mucinous neoplasms that are homogenously characterised with their matched peritoneal metastasis, demonstrating that the PSOGI 2016 peritoneal grade is an independent determinant of long-term outcomes (OS and DFS) for patients with PMP undergoing CRS + HIPEC with curative intent.

## Declarations

### Packages used for statistical analysis and graph generation

Statistical analysis: IBM SPSS Statistics, version 29.0.0.0. Survival graphs generated using: RStudio, version 4.4.1
